# Sometimes More is Too Much: A Rejoinder to the Commentaries on Greiff et al. (2015)

**DOI:** 10.3390/jintelligence5010006

**Published:** 2017-01-05

**Authors:** Samuel Greiff, Matthias Stadler, Philipp Sonnleitner, Christian Wolff, Romain Martin

**Affiliations:** 1 ECCS unit, University of Luxembourg, 11, Porte des Sciences 4366 Esch-sur-Alzette, Luxembourg; matthias.stadler@ur.de (M.S.); philipp.sonnleitner@uni.lu (P.S.); romain.martin@uni.lu (R.M.); 2 Lehrstuhl für Schulpädagogik, Universität Regensburg, 93053 Regensburg, Germany; 3 Organizational and Business Psychology, Technische Universität Darmstadt, 64289 Darmstadt, Germany

**Keywords:** complex problem solving, multiple complex systems, Tailorshop, reasoning, intelligence, validity, structural equation modeling

## Abstract

In this rejoinder, we respond to two commentaries on the study by Greiff, S.; Stadler, M.; Sonnleitner, P.; Wolff, C.; Martin, R. Sometimes less is more: Comparing the validity of complex problem solving measures. *Intelligence* **2015**, *50*, 100–113. The study was the first to address the important comparison between a classical measure of complex problem solving (CPS) and the more recent multiple complex systems (MCS) approach regarding their validity. In the study, we investigated the relations between one classical microworld as the initially developed method (here, the Tailorshop) with three more recently developed multiple complex systems (MCS; here, MicroDYN, Genetics Lab, and MicroFIN) tests. We found that the MCS tests showed higher levels of convergent validity with each other than with the Tailorshop even after reasoning was controlled for, thus empirically distinguishing between the two approaches. The commentary by Kretzschmar and the commentary by Funke, Fischer, and Holt expressed several concerns with how our study was conducted, our data was analyzed, and our results were interpreted. Whereas we acknowledge and agree with some of the more general statements made in these commentaries, we respectfully disagree with others, or we consider them to be at least partially in contrast with the existing literature and the currently available empirical evidence.

## 1. Introduction

The field of complex problem solving (CPS) has recently attracted considerable attention because of its relevance as an important 21st century skill and because of the many research questions that almost mystically surround CPS and that have yet to be empirically addressed. One recurring question that has kept researchers busy for decades is how to adequately measure CPS. In fact, researchers have developed a realm of diverse CPS tests that adhere to different assessment frameworks (i.e., classical microworlds [1], formal systems [2], and multiple complex systems [3]). By and large, evaluations and comparisons of these different approaches have been based more on opinion and conceptual arguments than on empirical studies of their properties [4,5,6]. In fact, for decades, this discussion has nearly ignored the need for an empirically established understanding of the construct validity of any CPS measure (for exceptions see e.g., [7,8]).

In an attempt to empirically compare two of the assessment frameworks, we investigated the relations between one classical microworld as the initially developed method (here, the Tailorshop) with three more recently developed multiple complex systems (MCS) tests (here, MicroDYN, Genetics Lab, MicroFIN) and published the results in Greiff et al. (2015) [9]. We also considered additional constructs (i.e., reasoning, school grades) in a sample of German University students. In sum, we found that the MCS tests showed higher levels of convergent validity with each other than with the Tailorshop even after reasoning was adjusted for, thus empirically distinguishing between the two approaches. The results were less clear with regard to predictive validity (criterion: school grades) as there were some indications that MCS showed higher levels of predictive and incremental validity than the Tailorshop but not consistently so. We interpreted the overall pattern of results as indicating that MCS had a higher level of construct validity than classical microworlds. To the best of our knowledge, the Greiff et al. (2015) study was the first to directly compare the classical microworld Tailorshop with MCS and, not surprisingly, both the results and their interpretation have re-sparked the long-standing [4,5] and sometimes heated debate on different CPS measurement approaches.^1^

This renewed interest is exemplified by two conceptually motivated commentaries on Greiff et al. (2015), to which we are responding here: One by André Kretzschmar, who was involved in developing one MCS-based test (i.e., MicroFIN) and one by Joachim Funke, Andreas Fischer, and Daniel Holt, who have been to varying degrees involved in developing and refining the Tailorshop microworld and some of the MCS measures. We acknowledge that the results reported in Greiff et al. (2015) and their interpretation have raised new questions, and we highly welcome and value the scientific discourse surrounding both our specific study and CPS as psychological construct on a more general level. In this rejoinder, we gratefully participate in and endorse a scientific discussion of this work *sine ira et studio*. While this rejoinder is not aimed at discussing every single aspect raised in the commentaries, we acknowledge and agree with some of the more general statements made in them (see minor points below), but we respectfully disagree with others or consider them to be at least partially in contrast with the existing literature and with the currently available empirical evidence (see major arguments below).

## 2. Minor Arguments

### 2.1. Choice of Intelligence Measure

The authors of both commentaries argued that the measure of intelligence chosen by Greiff et al. (2015) may have limited the validity estimates of intelligence. In our study, we used the Matrices subtest from the Intelligence Structure Test [10] to adjust the relations between the measures of CPS as well as the relations between CPS and students’ GPA in figural reasoning. Figural reasoning has repeatedly been shown to be the one facet of intelligence that most strongly conceptually and empirically overlaps with CPS (see [11,12]).

The commentaries, however, cited recent literature [12] (partially co-authored by some of the Greiff et al. authors) that convincingly demonstrated that a comprehensive operationalization of intelligence is able to explain considerably more variance in CPS performance than a rather specific operationalization of figural reasoning alone. They concluded that the potential underestimation of the relation between CPS and intelligence may have resulted in an overestimation of the common CPS variance, which should be taken into consideration.

We wholeheartedly agree that the measure used in our study did not represent a comprehensive operationalization of intelligence and that adjusting for such a measure might have resulted in somewhat different results with reduced correlations between all measures of CPS. However, it is not clear whether the pattern of correlations (as opposed to the general size of the correlations) actually would have changed, but this would be necessary for the arguments suggested by both commentaries to be valid. In fact, at this stage this contention remains the subject of mere speculation and calls for additional empirical research. Put differently, we do not know whether a comprehensive operationalization of intelligence would affect the MCS tests and the Tailorshop differently and whether adjusting for such a measure would, thus, change the correlational pattern between the different measures of CPS. Most of the cited studies on the relation between a broader measure of intelligence and CPS were published after Greiff et al., and answering both this research question and the ones the study was actually designed to answer would have gone far beyond the study’s scope. Thus, we were careful to use the term reasoning rather than intelligence in Greiff et al. (2015). However, we agree that a replication of our study using a comprehensive operationalization of intelligence would be worthwhile, and we strongly encourage researchers to take on this challenge.

### 2.2. Tailorshop, Only One Classical Microworld

Regarding the choice of the Tailorshop as a representative of classical measures of CPS, both commentaries mentioned that the use of only a single representative of classical microworlds reduced the broad class of classical CPS measures to a single task. This is definitely a limitation of our study, which we readily acknowledge and already discussed extensively in Greiff et al. (2015).

There were, however, some important considerations that led to our decision not to include multiple classical measures of CPS, and we would like to briefly reiterate them. First, Funke called the Tailorshop simulation the “drosophila of problem solving research” [4] and, correspondingly, the Tailorshop has been employed in the vast majority of studies that employed classical CPS measures. In fact, the Tailorshop is nowadays the only readily available classical CPS measure, whereas none of the other classical measures of CPS can, to our knowledge, be administered today due to compatibility issues and outdated software. Despite promising theoretical suggestions for a fuller conceptualization of CPS (e.g., [6]), a functioning test battery representing various facets of CPS or a more comprehensive classical measure of CPS is still missing and, to our knowledge, is nowhere to be found on the horizon of CPS research. In particular, research that relies on computer-based assessment (CBA) is constrained by the availability of technically flawless and running tools. Without even addressing the question of whether the usability of such tools has been demonstrated, it is to the very least essential to accommodate the needs of today’s digital natives in the context of CBA (for an empirical investigation on the usability of CPS measures see [13]). Although no information of the usability of any classical CPS measure exists, the Tailorshop still represents the “least problematic” and best-researched representative of classical CPS measures.

Again, we readily acknowledge that including only one classical CPS measure was a limitation of our study. However, as a fundamental prerequisite, other classical CPS measures would need to measure the same construct. Otherwise, the problems with these types of CPS measures would be rooted even deeper, and any study using the Tailorshop could not be generalized to either classical microworlds or to CPS as overarching construct [5]. As we discussed in Greiff et al. (2015) and as Funke et al. (p. 3) correctly stated in their commentary, “extant research suggests that classical microworlds, including the Tailorshop, show little empirical overlap.” Whereas we would not go as far as Funke et al. in suggesting that classical CPS measures do not measure a common construct, the lack of empirical evidence for the construct validity of most classical measures of CPS was another reason that we included only the Tailorshop in our study. To solve this problem, the authors of both commentaries recommended that other CPS measures, according to them other *classical* CPS measures, be included such as FSYS [8], LEARN! [14], or Powerplant [15]. Although a replication of our study that included these measures would be very interesting, it is important to note that not all of these are classical measures of CPS. In particular FSYS represents a formal system, as it does not emulate a real-world problem. Classical measures of CPS are designed to closely emulate real-world problem situations (e.g., running a business or even a whole city). Previous knowledge about similar problems (e.g., a business degree), therefore, provides a meaningful advantage toward solving the problem in these scenarios. Formal systems, on the other hand, simulate rather abstract problem situations that have no or only a fictitious relation (e.g., managing a forest with made-up trees and no further relation to real forest management; i.e., FSYS [8]) to real-world problems. Thus, the Tailorshop more closely resembles simulations such as Moro or Lohhausen (see [16]) than some of the microworlds suggested in the two commentaries. The theoretical and empirical distinctions between the three CPS assessment approaches are important for an adequate evaluation of theoretical claims and empirical findings. However, studies on the convergent validity between classical measures of CPS, formal systems, and MCS tests are still sparse.

Next to these rather minor arguments, which we generally agree with and which offer important directions for future research, both commentaries express several major arguments. We respectfully disagree, or only partially agree, with these arguments and will therefore respond to them in more detail.

## 3. Major Arguments

### 3.1. Inadequate Handling of the Tailorshop

One of the major criticisms in both commentaries refers to the application, scoring, and interpretation of the Tailorshop in Greiff et al. (2015). Kretzschmar and Funke et al. were concerned about the seeming lack of an exploration phase and the omission of a knowledge test, both of which they claim are essential parts of the Tailorshop. In addition, Funke et al. highlighted the problem of potential interdependences among the separate performance indicators of the Tailorshop and the need to consider the resulting autocorrelation. Regarding the interpretation of the resulting scores, Funke et al. further noted that using only a single microworld run compared with three MCS tasks with a total of 24^2^ runs may introduce a reliability problem at the level of manifest test scores and would not allow for a fair comparison between the MCS tasks and the Tailorshop.

Unfortunately and probably at the very source of the above-mentioned points, there is still no consistent recommendation for the application or the scoring of the Tailorshop despite its 30-year research history [11]. More specifically, a publication that lays out the exact structure of the Tailorshop for independent replications or that recommends any specific knowledge-test items is still missing (except for a highly extensive mathematical solution that has, to the best of our knowledge, not been used in any empirical study so far; see [17]). Rather, different publications feature quite diverse versions of the Tailorshop, including inconsistent descriptions of how to score and interpret the results even though all of them refer to them as “the Tailorshop.” Two rather recent studies [1,18] by Danner and colleagues (partially co-authored by the authors of the Funke et al. commentary) on the reliability and validity of the Tailorshop provide several recommendations though, which were explicitly used as a guideline for the handling of the Tailorshop in Greiff et al. (2015).

In fact, except for the knowledge test, in our study, we ran an almost identical version of the Tailorshop as Danner and colleagues, which included an exploration phase that preceded the control phase. We admit that the application of a standard exploration phase was not directly evident from reading Greiff et al. (2015) and not explicitly stated in there, but it seems deducible from the frequent and very specific references to the two studies. Of note, there are no version numbers or other ways to differentiate between different versions of the Tailorshop across empirical studies and different applications are used under the same label. The version of the Tailorshop used in our study (a very similar version also without a knowledge test is currently recommended by Funke and Holt at psychologie.uni-heidelberg.de/ae/allg/tools/tailorshop/) did not include a knowledge test because Danner and colleagues specifically recommended that only the control phase be scored because of reliability issues with the knowledge test. Thus, we disagree with the notion that Greiff et al. (2015) employed some kind of exotic version of the Tailorshop as, very much to the contrary, a version recommended by some of the authors of the commentaries was used.

Regarding the interdependences among the separate performance indicators of the Tailorshop, Funke et al. argued in their commentary that by ignoring the autocorrelation of the performance indicators, we artificially increased the measure`s reliability, which in turn led to an underestimation of the validity coefficients for the Tailorshop due to a reduced correction for attenuation. We agree that this would be a plausible argument if the autocorrelation had actually been ignored. However, Danner and colleagues ([18] p. 228) provided specific recommendations for how to best score the Tailorshop and stated that “the changes of the company values after each simulated month may be taken as performance indicators for the Tailorshop simulation” because “there is no [autocorrelation] between the changes of the company values” ([18] p. 227) as already described previously by Funke [19]. The empirical appropriateness of this approach is also demonstrated empirically in this paper [18]. As these are exactly the performance indicators we used in Greiff et al. (2015), it seems peculiar that Funke et al. would criticize us for following their own recommendations. Their criticism is thus either the result of a misunderstanding or should be directed at their own work, which then failed to provide an acceptable scoring for the Tailorshop in more than 30 years of research.

In addition to their criticism of the scoring of the Tailorshop, Funke et al. seem to imply that an aggregated score from multiple runs of the Tailorshop might provide a better comparison with the MCS tests, which consist of multiple small microworlds (p. 2). Of note, multiple runs of the Tailorshop are impracticable due to the unrealistically high testing time (1 h, if not considerably more), are implausible due to learning effects that would severely reduce the measure’s reliability (for an potential use of parallel forms that reduce this problem see [20]), and have little to no theoretical or empirical foundation. On the contrary, the single run of one very large complex problem is at the heart of the construction rationale of the classical CPS measures [3]. Any suggestions about potential results of multiple runs of the Tailorshop must therefore remain purely speculative.

This is exactly the advantage and the main rationale behind the MCS measures. In response to some of the obvious psychometric problems of classical CPS measures (see previous point), MCS measures were designed to be short enough to allow for the assessment of multiple tasks, which are independent of each other and are, thus, less prone to learning effects [3]. The resulting score, thus, contains the information of multiple independent measures and is therefore considerably more reliable than a score that is the result of a single large complex system. Funke et al. do not specify how they would suggest amending this issue but imply that not addressing it represents an unfair treatment of the Tailorshop. We do agree with Funke et al., however, that comparing an aggregated measure of all three MCS measures with the Tailorshop would be inadequate. Aggregated into one factor, the three MCS measures would represent a higher level of abstraction than the Tailorshop (i.e., a second-order factor), which is why we refrained from doing so in Greiff et al. (2015). Rather, we chose to compare the validity of each MCS measure with the Tailorshop separately.

Finally, Funke et al.’s criticism that the resulting MicroDYN factor (we assume they combined MicroDYN and the Genetics Lab in this criticism) might be a “bloated specific” due to the very high similarity between the tasks would be true for every construct that is measured by applying a well-defined construction rationale (e.g., also for figural matrices tasks that appear to be quite similar but rely on different sets of construction rules [21]). Only the variation and addition of such rules leads to varying task difficulties. However, tasks such as matrices are among the best-justified psychometric measures and are rather unlikely to constitute a bloated specific. Following this line of thought, Stadler, Niepel, and Greiff [22] demonstrated that tasks within one of the MCS measures, MicroDYN, can be constructed based on a set of six well-defined rules that fully determine their difficulty. Therefore, it seems implausible that MicroDYN tasks would constitute a bloated specific without further empirical evidence addressing this issue.

In summary, we respectfully disagree with the very general criticisms of our handling of the Tailorshop expressed in both commentaries. Our application, scoring, and interpretation procedures were based on the latest publications on the Tailorshop, which may be at odds with other publications that used the Tailorshop. However, this seems more like a general issue with the Tailorshop than with our paper.

### 3.2. Inadequate Inclusion of MicroFIN

Both commentaries argued that the specific version of MicroFIN we included was inadequate, mainly due to the reduced number of only two MicroFIN tasks. We wholeheartedly agree and consider this a shortcoming of Greiff at al. (2015) and also of Greiff et al. (2014) [23], which relied on the same data but addressed a different research question. Of note, both papers acknowledged this shortcoming, discussed it in detail, and provided a number of arguments for the inclusion of MicroFIN albeit in a limited and rather brief version (cf. [9,23]).

However, the two commentaries drew quite contrasting conclusions about MicroFIN, and their interpretations disagreed. Funke et al. declared the 2-task version of MicroFIN the “surprise winner” (p. 4) with respect to predictive validity. They did so on the basis of absolute differences in correlation coefficients with school grades in the range of lower than 0.05 (e.g., 0.22 vs. 0.19 or 0.33 vs. 0.31; cf. Table 2 in Greiff et al., 2015; see [24], for variability in correlation coefficients). From this, the authors concluded that the entire idea of MCS, which propagates the need for several independent CPS tasks, might be an unnecessary one as only two MicroFIN tasks were enough to outperform MicroDYN (10 tasks) and the Genetics Lab (12 tasks). Kretzschmar, on the other hand, stressed the need for a longer (i.e., more tasks) and presumably more reliable version of MicroFIN. He then argued that this would allegedly lead to a higher correlation between Tailorshop and MicroFIN and thus questioned Greiff et al.’s (2015) argument for higher convergent coefficients between the MCS tasks than between any MCS task and the Tailorshop. In his argument, however, Kretzschmar did not consider that in the envisaged scenario the relations between MicroFIN and the two other MCS measures would also increase, leading to even more consistency between the three MCS tests (MicroFIN had the lowest relations with MicroDYN and the Genetics Lab; cf. Table 2 in Greiff et al., 2015) and, further, that the issue of reliability on the level of latent modeling is unlikely to be a driving factor of latent correlations even to begin with.

Although we endorse the limitations associated with the two MicroFIN tasks, we note that both arguments are based on speculation and cannot be confirmed or rebutted without further empirical study. We invite the authors of the two commentaries to conduct empirical investigations of their propositions. However, in the absence of this, we need to rely on the little empirical evidence there is. Neubert, Kretzschmar, Wüstenberg, and Greiff [25] published the only article that employed a longer version of MicroFIN with five tasks. In correlated trait-correlated methods minus 1 (CTC(M-1) [26]) models, the average specificity across all five MicroFIN tasks is reported as 0.58 (cf. Table A1 in Neubert et al.) in a model with MicroDYN as the reference method. In a similar model, again with MicroDYN as the reference method, the two MicroFIN tasks employed in Greiff et al. (2015) had an average specificity of 0.53 (cf. Table 2 in Greiff et al., 2014). Put differently, the latent MicroFIN factor showed a comparable overlap/distinction with MicroDYN in both data sets irrespective of the number of MicroFIN tasks.

Thus, we cannot see how either the data reported in Greiff et al. (2015) or the (admittedly little) other empirical evidence suggests that the reliability of MicroFIN or the overall pattern of results might be distorted to an extent that would render our original conclusions invalid, and we felt that the commentaries did not offer clear and sufficiently reasoned conclusions here either.

### 3.3. Inadequate Statistical Analyses and Interpretations

Finally, Kretzschmar in particular discussed issues with Greiff et al.’s (2015) statistical analyses and offered several alternative interpretations of the results. We acknowledge that there are various ways to analyze any data, some of which certainly provide interesting new insights. However, we insist that the analyses we chose were adequate insofar as they allowed us to answer the research questions outlined in our study.

To answer Research Question 1 (RQ1), whether the correlations between the different MCS tests were higher than those between the MCS tests and the classical CPS measure, we compared a restricted model with equal correlations between all CPS measures and a less restricted model with equal correlations between the MCS tests and allowed for different values in the correlations between the MCS tests and the Tailorshop. Kretzschmar argued that a lack of fit of the less restricted model (after adjusting for reasoning) might have affected our findings and questioned the validity of our approach. While we do not agree with his criticism,^3^ we gladly conducted additional analyses that provided further support for the interpretations made in Greiff et al. (2015). We based these analyses on the intercorrelations between all manifest indicators of the four CPS tests. If a lack of model fit had biased our original results, as Kretzschmar suggested, we should find a deviating pattern when using manifest variables instead of latent factors. We calculated hetereotrait-monotrait ratios between all CPS tests [27]. This means that we related the strength of the correlation between indicators of two different CPS tests to the strength of the correlation between indicators of the same CPS test. We found that the ratios within the MCS tests (0.60 to 0.76) were higher than the ratios between the MCS tasks and the Tailorshop (0.24 to 0.45), and these ratios remained similar when reasoning was controlled for (0.51 to 0.71 vs. 0.19 to 0.41). This indicates convergent validity for the MCS tests as compared with the Tailorshop. Even when the analytical model was free from any restricting assumptions, we found results that were similar to those from the original analyses and to the latent correlations reported by Greiff et al. (2015; Table 2).

Funke et al. also questioned the analytical approach chosen to answer RQ1 because, in this comparison, there was no way the Tailorshop could have been more valid than the MCS tests since there was no other test in its group (classical microworlds) that it could correlate with. This appears to be the result of a misunderstanding. As outlined throughout the Greiff et al. paper, our hypothesis was that the MCS tests would be more closely related to each other than to the Tailorshop and would, thus, show higher convergent validity. If there is a CPS construct and MCS tests capture it more reliably than the Tailorshop, we would expect higher and more consistent correlations between MCS tests than between any MCS tests and the Tailorshop. On the basis of our approach, this hypothesis could have been rejected by a nonsignificant difference between the two models suggesting equal correlations between all CPS measures.

Furthermore, both commentaries emphasized the importance of adjusting our results for reasoning (or better, a comprehensive measure of intelligence). Concerning RQ2, comparing the validity of the MCS tests and the Tailorshop in predicting students’ GPAs before and after adjusting for reasoning, the authors of the commentaries pointed out how weak (statistically nonsignificant) our results were after reasoning was controlled for. They interpreted this finding in the light of our conclusion that the construct validity of MCS tests might somehow be superior to the construct validity of the Tailorshop. This reasoning is based on contradictory assumptions. First, they correctly assumed that intelligence is an important control variable because it predicts the same criteria as CPS measures do and that the reason for this is that CPS tests can be subsumed under the overarching construct of intelligence as can reasoning [28]. Second, they were surprised that, after reasoning was adjusted for, the differences between MCS and Tailorshop decreased. If the constructs of CPS and reasoning exhibit some overlap, and MCS tests are valid measures of the construct of CPS, then it logically follows that the correlations between MCS tests and reasoning should be particularly high (which is what we and [29] found). If MCS tests are more valid measures of the construct of CPS than the Tailorshop is, then their overlap with reasoning should be higher than the overlap between the Tailorshop and reasoning (which is what we found). This means that adjusting the MCS tests and the Tailorshop for reasoning has a stronger effect on the better test of CPS (here, presumably the MCS tests) because it shares more variance with reasoning and is therefore particularly affected by controlling for reasoning.

In summary, we agree that additional analyses leading to potentially different interpretations are possible, and we were thus careful about how we phrased our results. However, we still believe that the methods used and the interpretation of our results were adequate for answering the intended research questions and led to important and long-overdue progress in the field of CPS. If other researchers would like to explore different analyses or research questions with the data, we would be happy to provide the data for reanalyses.

## 4. Outlook

As the two commentaries demonstrate, there is considerable interest in the field of CPS. They also reveal that the field is in serious need of further empirical research and a strong theoretical foundation. Only by summarizing existing research [29] and providing new empirical evidence can we answer the pressing research questions that have now been highlighted by several recent comments and opinion papers (see [4,5]). There is, therefore, a dire need for original theoretical and empirical research to move the field of CPS research along. A lack of theoretical and empirical progress due to a persistent and unproductive debate that has been based on opinions rather than facts may have already led to the decline in interest in CPS that occurred in the late 1990s.

To avoid a repeated decline in research on CPS, there are several steps that are necessary to help the field of CPS research move forward. First, the theoretical basis of CPS needs to be strengthened by finally defining the cognitive processes involved in solving complex problems. Moreover, researchers need to determine whether CPS can be understood as a broad construct. If so, what are the facets that determine this construct, and is there still an overarching general factor of CPS? To our knowledge, MCS-based measures are currently those that show the closest link to definitions of CPS without being confounded with other constructs such as prior knowledge [3]. Obviously, this does not preclude or defy the need for further advances in the assessment of CPS, be it with MCS tests or classical microworlds.

Alongside these theoretical and measurement issues, several empirical questions demand answers. As highlighted throughout this rejoinder, a study investigating the convergence of the three assessment approaches proposed for CPS is still missing. Such a study could provide important information on the generalizability of previous CPS research. In addition, Funke et al. are correct in questioning whether school grades are in fact the ideal complex real-world behavior to validate CPS measures. We are looking forward to research on different real-world indicators of CPS (e.g., indicators of job success or even political skills) and how they are related to CPS task performance. Finally, we see a great need for the development of new CPS assessment instruments in the classical tradition that do not suffer from the psychometric problems of earlier classical measures of CPS [3]. If more really is more, that is, if classical measures of CPS indeed capture aspects of complex problem solving ability that extend MCS, then sound measurement instruments are a prerequisite to highlighting what these aspects are. Without such developments, the potential benefits of more complex measures of CPS will always remain speculative, and pointing fingers at simpler but psychometrically sound (at the very least in terms of providing consistent measures) tools such as those developed in the MCS approach will become meaningless.

Thus, we clearly and readily acknowledge the need for further research on the topic, and we endorse all efforts that are moving in this direction. However, speculation about the possible implications of shortcomings that are extensively discussed in original articles without considering the overall pattern of results is unlikely to add any lasting substance to the debate.

## Footnotes

1 For an interesting read on an excellent example of this early discussion that includes a direct and strong criticism of the Tailorshop, please consult the article by Funke (1983) that dates back over 30 years ago (in German only) [10].

2 Instead of 16 GL items, hence, 28 MCS tasks in total mentioned by Funke et al., we used only 12 GL items, hence, 24 MCS tasks in total.

3 Kretzschmar correctly pointed out that some heterogeneity exists within MCS tests as indicated by some significantly different relations of MCS tests with each other. However, all correlations within MCS tests were numerically higher (0.62-0.73) than all correlations with Tailorshop (0.25-0.43; Table 2 in Greiff et al., 2015). Furthermore, the tests were grouped a priori on the basis of their design features. Therefore, we do not think that heterogeneity within the MCS tests severely limited the suitability of our analytical approach.
